# The Impact of Hypothyroidism on Diabetes Mellitus and Its Complications: A Comprehensive Review

**DOI:** 10.7759/cureus.40447

**Published:** 2023-06-15

**Authors:** Shree Laya Vemula, Saikumar Aramadaka, Raam Mannam, Rajagopal Sankara Narayanan, Arpit Bansal, Vishnu R Yanamaladoddi, Sai Suseel Sarvepalli

**Affiliations:** 1 Department of Internal Medicine, Anam Chenchu Subba Reddy (ACSR) Government Medical College, Nellore, IND; 2 Department of Internal Medicine, Narayana Medical College, Nellore, IND; 3 Department of General Surgery, Narayana Medical College, Nellore, IND; 4 Department of General Surgery, Narayana Medical College and Hospital, Nellore, IND

**Keywords:** type 2 diabetes mellitus, hypothyroidism and type 2 diabetes mellitus, thyroid disorder, endocrine disorders, diabetic microvascular complications

## Abstract

Diabetes mellitus (DM) is one of the most prevalent metabolic disorders in the world and is characterized by excessive blood glucose levels, which lead to deranged carbohydrate, protein, and lipid metabolisms. At its core, DM is an impairment of insulin metabolism, leading to a plethora of clinical features. The thyroid gland is another vital cog in the wheel of the endocrine system, and the hormones synthesized by it are heavily involved in the control of the body’s metabolism. Hypothyroidism is a state in which thyroid hormones are deficient due to various factors and is characterized by a metabolically hypoactive state. Together, insulin, implicated in DM, and thyroid hormones engage in an intricate dance and serve to regulate the body’s metabolism. It is imperative to explore the relationship between these two common endocrine disorders to understand their clinical association and mold treatments specific to patients in which they coexist. Both type 1 diabetes mellitus (T1DM) and type 2 diabetes mellitus (T2DM) have been shown to have an increased association with hypothyroidism, especially in patients with risk factors including female sex, hyperlipidemia, obesity, and anemia. This review also explores DM’s macrovascular and microvascular complications and their association with hypothyroidism. It is of great use to screen for hypothyroidism in diabetic patients. Specific protocols, especially for patients at an elevated risk, provide improved quality of life to patients affected by this highly prevalent disease.

## Introduction and background

Diabetes mellitus (DM) is a debilitating disease caused by dynamic interactions between genes, environment, and other lifestyle factors such as obesity and a sedentary lifestyle [1]. Diabetes mellitus is characterized by excessive blood glucose levels caused by suboptimal or total deficiency of insulin secretion by the pancreas, known as type 1 diabetes mellitus (T1DM), or body cells’ poor utilization of insulin, known as type 2 diabetes mellitus (T2DM), showing its effects on carbohydrate, fats, and protein metabolism [2,3]. According to the data presented by the International Diabetes Federation (IDF) in 2021, there were 537 million diabetics worldwide, which is anticipated to rise to 643 million by 2030 and 783 million by 2045 [4]. In addition to the pancreas, the thyroid gland affects the body’s metabolism. Hormones secreted by the thyroid gland regulate carbohydrate metabolism and insulin secretion. Both these hormones are closely linked to one another. Hence, any change in one hormone’s level might affect another hormone’s effectiveness [2]. This association between thyroid illnesses and diabetes mellitus dates back to 1979 [5]. Individuals with type 2 diabetes mellitus seemed to have a significantly greater prevalence of thyroid disorders [6,7]. Researchers have looked into the association between diabetes and thyroid dysfunction and found that hypothyroidism is common in patients with diabetes, with prevalence rates ranging from 4.8% to 31.4% [8]. Thyroid disorders tend to be predominant among female diabetics [9]. Diabetic patients with longstanding hyperlipidemia, obesity, and anemia are at higher risk of having underlying hypothyroidism [9]. The clinical correlation between these two prevalent endocrine illnesses tends to be high, analogous to the impact of hypothyroidism on the development of diabetic complications. This review article aims to ascertain the prevalence and pathogenesis involved in the development of thyroid dysfunction, precisely hypothyroidism in patients with type 2 diabetes mellitus, and its treatment and explore the effects of hypothyroidism on the subsequent development of diabetic complications.

## Review

Methodology

We conducted a review by searching the databases of PubMed and Google Scholar. We obtained studies published between January 1, 1980, and December 31, 2022. The keywords used to identify relevant articles include type II diabetes mellitus and hypothyroidism. The studies were selected to evaluate the prevalence of hypothyroidism in type 2 diabetes mellitus and identify which complications were more common among hypothyroid patients with type 2 diabetes mellitus. A total of 20 articles were identified, which included data from a total of 116 studies.

Type 2 diabetes mellitus and hypothyroidism

Type 2 diabetes mellitus (T2DM) occurs due to insulin’s inability to enhance the absorption and utilization of glucose in peripheral tissues (muscle, adipose tissue, and liver) and insulin resistance alongside a progressive reduction of beta cell insulin production [10]. Approximately 537 million adults had diabetes in 2021, of whom 90% had T2DM. Nair et al. [9] conducted a study involving 1,152 patients in India in 2018 on the prevalence and associations of hypothyroidism in Indian patients with T2DM. They concluded that one-tenth of Indian patients with T2DM showed clinical hypothyroidism and another 5% showed subclinical hypothyroidism (SCH). Although T2DM is typically diagnosed in older adults, it is becoming more common in children, adolescents, and younger adults due to rising obesity, inactivity, and poor diet [4]. An increased risk of hypothyroidism in T2DM is attributed to female sex [9], older age, obesity, and thyroid peroxidase antibody (TPO Ab positive) [11-13].

**Figure 1:**
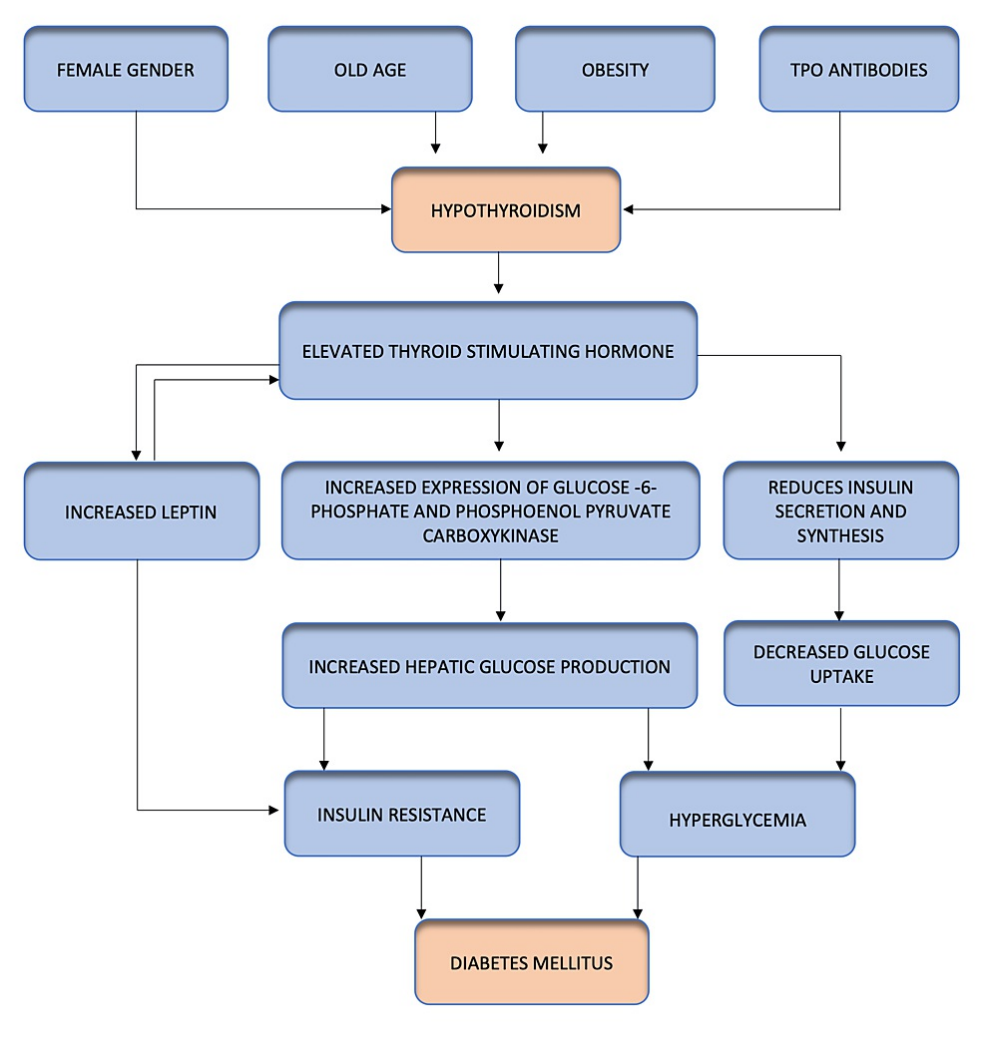
Pathophysiology of hypothyroidism-mediated diabetes mellitus TPO: thyroid peroxidase Image credits: Dr. Shree Laya Vemula

At early stages, hyperinsulinemia can compensate for insulin resistance for proper glucose uptake and utilization in the peripheries. Later, beta cells cannot tolerate chronic hyperinsulinemia, which might result in the onset of postprandial hyperglycemia leading to overt T2DM [14]. The reason for the elevated thyroid-stimulating hormone (TSH) in people with diabetes is uncertain, as it may result from complex interdependent interactions. Previous studies suggested that insulin resistance, hyperglycemia, and leptin influence the level of serum thyroid-stimulating hormone (TSH) [15]. Leptin, by modulating the hypothalamic-pituitary-thyroid axis via the Janus activating kinase (JAK)-2 or signal transducer and activator of transcription (STAT)-3 factor, tends to increase serum TSH levels in numerous diabetic patients [16], and in turn, TSH increases leptin secretion from adipose tissues. TSH and leptin altogether play a significant role in the metabolism of hepatic glucose by acting at the mRNA level, resulting in increased expression of glucose-6-phosphate and phosphoenolpyruvate carboxykinase (PEPCK), which has stimulatory effects on hepatic glucose production [17-19]. Additionally, TSH increases serum blood glucose levels by decreasing insulin secretion and its synthesis from pancreatic beta cells [18]. The pathogenesis involved in hypothyroidism-mediated T2DM is discussed in Figure 1.

Chaker et al. [19] conducted a population-based prospective cohort study in 2016 at Rotterdam with 8,452 participants and concluded that a higher likelihood of diabetes and the development of prediabetes into diabetes were associated with lower free thyroxine 4 (FT4) levels (hazard ratio (HR): 0.91, 95% confidence interval (CI): 0.86-0.97) and higher TSH levels (HR: 1.32, 95% CI: 1.06-1.64). Walia et al. [20] performed a meta-analysis in 2022 involving 36,500 patients and concluded that T2DM patients are at increased risk of developing subclinical hypothyroidism (odds ratio (OR): 1.88, 95%CI: 1.33-2.66). In contrast to these studies, Menon et al. [21] conducted a cohort study with 1,180 patients consisting of both diabetic and nondiabetic individuals. A community cohort with 986 patients and a hospital cohort with 194 patients were included in this study. They performed thyroid function tests and thyroid autoantibodies tests in all subjects. They concluded that the prevalence of thyroid disorders is identical in diabetic and nondiabetic individuals. The studies exploring the association between hypothyroidism and the development of T2DM are summarized in Table 1.

**Table 1:** Studies exploring the association between hypothyroidism and the development of type 2 diabetes mellitus T2DM: type 2 diabetes mellitus, Pre-DM: pre-diabetes mellitus, TSH: thyroid-stimulating hormone, FBS: fasting blood sugar, HbA1c: hemoglobin A1c

Study	Design	Number of participants	Population	Main conclusion
Tamez-Pérez et al. (2012) [22]	Observational study	5,161	T2DM individuals and nondiabetic individuals	Hypothyroidism and T2DM are linked to one another.
Distiller et al. (2013) [6]	-	922	T2DM individuals	The occurrence of hypothyroidism is higher among T2DM individuals.
Raghuwanshi et al. (2015) [23]	-	80	T2DM patients in Bhopal	Thyroid disorders are prevalent in patients with T2DM.
Chaker et al. (2016) [19]	Population-based prospective cohort study	8,452	T2DM patients in Rotterdam	Prediabetic individuals with low thyroid function are at increased risk of developing diabetes.
Telwani et al. (2017) [24]	Case-control study	200	Diabetic and nondiabetic individuals	Thyroid dysfunction is prevalent among diabetic patients.
Menon et al. (2019) [21]	Cohort study	1,180	Diabetic and nondiabetic individuals from the same locality in India	The prevalence of thyroid disorders is the same in diabetic and nondiabetic individuals.
Alsolami et al. (2018) [25]	Case-control study	242	Diabetic and nondiabetic individuals in Saudi Arabia	Hypothyroidism is more prevalent among T2DM patients, and its risk can be reduced in T2DM patients by adequate glycemic control.
Al-Rubaye (2019) [26]	Cross-sectional study	500	T2DM patients in Iraq	Diabetic individuals with poor glycemic control are at higher risk of developing hypothyroidism.
Huang et al. (2020) [27]	Community-based population study	3,986	Residents aged 26-76 years in Beijing	The coexistence of thyroid disorders is high in patients with T2DM and pre-DM.
Khassawneh et al. (2020) [28]	Case-control study	998	T2DM patients in Jordan	Thyroid disorders are more prevalent in T2DM patients.
Vamshidhar et al. (2020) [29]	Cross-sectional study	50	T2DM patients in India	Thyroid disorders are more prevalent in patients with T2DM, and a positive correlation of TSH with FBS and HbA1c was seen.
Walia et al. (2022) [20]	Meta-analysis	36,500	Analysis of 62 studies from various databases from 2005 to 2022 involving T2DM and hypothyroid patients	Patients with T2DM are at increased risk of developing subclinical hypothyroidism.

Hypothyroidism and diabetic complications

Individuals with type 2 diabetes mellitus have a higher prevalence of hypothyroidism, and patients with both conditions have a higher prevalence of microvascular complications [5]. Microvascular complications include diabetic nephropathy, diabetic retinopathy, and diabetic peripheral neuropathy; macrovascular complications include peripheral artery disease. Studies showed that diabetic individuals with hypothyroidism had reduced glomerular filtration rate, renal blood flow, and cardiac output and increased peripheral vascular resistance. All these factors contribute to the development of renal complications in patients with both conditions [30,31]. Patients with both of these conditions tend to have a lower insulin-like growth factor-1 (IGF-1), which is responsible for maintaining retinal vasculature. IGF-1 is lowered in hypothyroid rats, leading to thinner and smaller retinas [32], contributing to diabetic retinopathy. Hypothyroidism causes Schwann cells’ metabolic disorders, leading to its segmental demyelination, causing diabetic peripheral nephropathy [33]. According to researchers, hypothyroidism may cause increased arterial stiffness and endothelial dysfunction, contributing to peripheral arterial disease (PAD) [34].

Chen et al. [35] conducted a cross-sectional analysis in Taiwan. A total of 588 patients with T2DM were included, and their serum thyroxine and TSH were measured. Compared to euthyroid subjects, hypothyroid subjects had a higher prevalence of diabetic nephropathy (OR: 3.15, 95% CI: 1.48-6.69), but not diabetic retinopathy (OR: 1.15, 95% CI: 0.59-2.26). In contrast, Kim et al. [36] performed a retrospective study in 2011 in Korea. Overall, 489 patients with diabetic retinopathy were assessed for their severity and serum TSH levels. They concluded that patients with severe diabetic retinopathy had subclinical hypothyroidism (OR: 2.086, 95% CI: 1.010-4.307, P=0.047).

Gao et al. [37] performed a study in China in 2014. The serum TSH, free T4, and free T3 levels of 194 diabetes mellitus patients were assessed and subjected to diabetic microvascular complications screening (nephropathy, retinopathy, and neuropathy). They concluded that in diabetic patients with upper normal levels of TSH, no association is found between subclinical hypothyroidism and the development of microvascular complications such as retinopathy (OR: 0.753, 95% CI: 0.203-2.793, P=0.671), nephropathy (OR: 1.312, 95% CI: 0.304-5.659, P=0.716), and neuropathy (OR: 0.591, 95% CI: 0.156-2.232, P=0.428).

Han et al. [5] conducted a meta-analysis of 36 studies from various databases from 1980 to 2014 with 13,740 T2DM patients. They concluded that the development of diabetic complications such as nephropathy (OR: 1.74, 95% CI: 1.34-2.28), diabetic retinopathy (OR: 1.42, 95% CI: 1.21-1.67), peripheral arterial disease (OR: 1.85, 95% CI: 1.35-2.54), and diabetic peripheral neuropathy (OR: 1.87, 95% CI: 1.06-3.28) are affected by subclinical hypothyroidism. In contrast to this, Walia et al. [20] also conducted a meta-analysis of 62 studies from various databases from 2005 to 2022 with 36,500 T2DM and hypothyroid patients and concluded that diabetic nephropathy was more prevalent in hypothyroid patients (OR: 3.31, 95% CI: 1.56-7.02), but not diabetic peripheral neuropathy (OR: 1.11, 95% CI: 0.62-2.00) and cardiovascular diseases (OR: 1.68, 95% CI: 0.95-2.97). The studies exploring the effect of hypothyroidism on the development of diabetic complications are summarized in Table 2.

**Table 2:** Studies exploring the effect of hypothyroidism on the development of complications of type 2 diabetes mellitus T2DM: type 2 diabetes mellitus, TSH: thyroid-stimulating hormone

Study	Design	Number of participants	Population	Main conclusion
Chen et al. (2007) [35]	Cross-sectional analysis	588	T2DM patients in Taiwan	There was an increased risk of nephropathy and cardiovascular events, not retinopathy.
Kim et al. (2011) [36]	Retrospective study	489	T2DM patients who attended diabetes clinics between 2001 and 2007 in Korea	There was an increased risk of diabetic retinopathy.
Furukawa et al. (2014) [38]	Cross-sectional analysis	414	T2DM patients in Japan	There was an increased risk of diabetic nephropathy.
Gao et al. (2014) [37]	-	194	T2DM patients in China	In diabetic patients with upper normal levels of TSH, no association was found between subclinical hypothyroidism and the development of microvascular complications.
Han et al. (2015) [5]	Meta-analysis	13,740	Analysis of 36 studies from various databases from January 1, 1980, to December 1, 2014, of T2DM and hypothyroid patients	Hypothyroidism increases the risk of developing diabetic nephropathy and diabetic peripheral neuropathy.
Jose et al. (2020) [39]	Case-control study	384	T2DM with nephropathy and without nephropathy in India	The independent risk factor for the development of diabetic nephropathy is hypothyroidism.
Golwalkar et al. (2021) [40]	Cross-sectional analysis	100	T2DM patients in India	There was an increased risk of diabetic retinopathy and diabetic nephropathy, but not cardiovascular diseases.
Allam et al. (2021) [41]	Cross-sectional study	300	Diabetic peripheral neuropathy patients in Cairo	The prevalence of diabetic peripheral neuropathy is higher in patients with hypothyroidism.
Walia et al. (2022) [20]	Meta-analysis	36,500	Analysis of 62 studies from various databases from 2005 to 2022 in T2DM and hypothyroid patients	There was an increased risk of diabetic nephropathy in T2DM and no significant risk of developing diabetic peripheral neuropathy and cardiovascular diseases.

Treatment of hypothyroidism in diabetes mellitus patients

Hypothyroidism in T2DM patients is treated with oral levothyroxine 4 (L-T4) daily. The American Thyroid Association (ATA) and the European Thyroid Association (ETA) both recommend treatment when serum TSH levels are >10 mU/L [42]. Insulin sensitivity and fasting hyperinsulinemia are significantly improved by L-T4 [43]. After the beginning of L-T4 therapy, serum TSH levels should be rechecked in two months, and dosage adjustments must be made. After initiating L-T4 medication for patients with SCH, serum TSH should be checked at least once a year [42]. Most individuals aim to maintain a stable serum TSH level in the lower half of the recommended ranges (0.4-2.5 mU/L).

Limitations

There are a few limitations to this review article. Several research articles found on PubMed and Google Scholar were used to collect data and varied in sample size and methodology. Other databases were not used. There is also a lack of specific diagnostic interventions, which should be further explored. There is also a disparity in data pertaining to certain complications associated with these two conditions, with a few studies contraindicating each other’s findings.

## Conclusions

This article explores the relationship between hypothyroidism and diabetes mellitus, two common endocrine disorders with massive clinical implications. This review article establishes a correlation between diabetes mellitus and hypothyroidism, which are associated with decreased long-term quality of life and morbidity due to microvascular and macrovascular complications. We believe that this article can help raise awareness among clinicians in correlating these two diseases by elaborating on the pathophysiological role of how hypothyroidism develops in patients affected by both type 1 and type 2 diabetes mellitus, the various complications associated with both conditions, and the treatment modalities available. We conducted a thorough analysis of the complications, providing a comprehensive overview of the most common complications that should be taken into consideration when managing patients with coexisting diseases. Early identification of hypothyroidism in patients with diabetes mellitus (DM) through a targeted screening protocol can significantly reduce the occurrence of complications by ensuring prompt and appropriate treatment. Further research into the association between hypothyroidism and DM, along with specific treatment, can help create a tailor-made protocol for management.
